# Progress towards completing the mutant mouse null resource

**DOI:** 10.1007/s00335-021-09905-0

**Published:** 2021-10-26

**Authors:** Kevin A. Peterson, Stephen A. Murray

**Affiliations:** The Jackson Laboratory, Bar Harbor, ME USA

## Abstract

The generation of a comprehensive catalog of null alleles covering all protein-coding genes is the goal of the International Mouse Phenotyping Consortium. Over the past 20 years, significant progress has been made towards achieving this goal through the combined efforts of many large-scale programs that built an embryonic stem cell resource to generate knockout mice and more recently employed CRISPR/Cas9-based mutagenesis to delete critical regions predicted to result in frameshift mutations, thus, ablating gene function. The IMPC initiative builds on prior and ongoing work by individual research groups creating gene knockouts in the mouse. Here, we analyze the collective efforts focusing on the combined null allele resource resulting from strains developed by the research community and large-scale production programs. Based upon this pooled analysis, we examine the remaining fraction of protein-coding genes focusing on clearly defined mouse–human orthologs as the highest priority for completing the mutant mouse null resource. In summary, we find that there are less than 3400 mouse–human orthologs remaining in the genome without a targeted null allele that can be further prioritized to achieve our overall goal of the complete functional annotation of the protein-coding portion of a mammalian genome.

## Introduction

Animal models, including mouse knockouts, play an instrumental role in advancing our understanding of how disruption of normal gene function relates to human disease. Traditionally, much of this work focuses on a relatively small number of conserved genes and pathways, reflecting a common tendency for investigators to incrementally build upon existing knowledge. Often these advancements reflect the critical importance of specific genes to human disease (Dolgin 2017); however, the “lamppost effect” greatly limits the opportunity for novel discoveries of gene function (Stoeger et al. 2018), and ultimately restricts the development of new therapeutic avenues (Oprea et al. 2018). This effect is strongly driven by the availability of tools to interrogate gene/pathway functions (Edwards et al. 2011), including mouse mutants (Stoeger et al. 2018). Further, this problem is compounded by poor public availability and/or limited phenotypic characterization of many of these mutant mouse lines. Herein, we summarize our progress towards completion of the mutant mouse null resource that promises to advance our understanding of novel genes and pathways, while reducing the research barriers to entry for individual investigators.

**Fig. 1:**
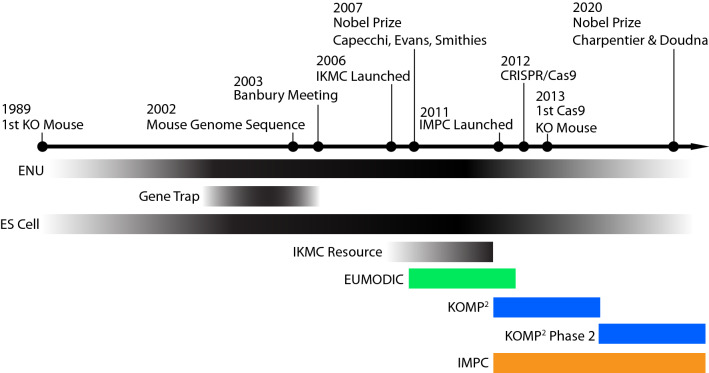
Timeline highlighting major milestones enabling complete functional annotation of the protein-coding fraction of the mouse genome. Technological advances that made possible the large-scale generation of mutant mouse resources are shown in black. Multi-institutional collaborative programs (e.g., European Mouse Disease Clinic; EUMODIC, and Knockout Mouse Phenotyping Program; KOMP^2^) implemented these advancements to perform high-throughput animal model production and systematic broad-based phenotyping. This collective work has grown to include additional international sites that have been centralized under the International Mouse Phenotyping Consortium (IMPC) to coordinate animal production, phenotyping, and data dissemination

Over the past 20 years, tremendous progress has been made in generating a complete mutant mouse resource covering the roughly 23,000 protein-coding genes in the mouse genome. These efforts, spurred by the publication of the draft sequence of the mouse genome in 2002 (Mouse Genome Sequencing et al. 2002), reflect the consensus view that the mouse is the premier model of mammalian biology and the accumulation of technological innovations in mouse genetics presented a viable path towards functional annotation of the entire mouse genome (Fig. 1; Austin et al. 2004; Auwerx et al. 2004; Birling et al. 2021; Capecchi 1989; de Angelis et al. 2015; International Mouse Knockout et al. 2007; Jinek et al. 2012; Wang et al. 2013). This technological convergence includes the widespread adoption of mouse embryonic stem cells (mESCs) as a platform for gene targeting and innovations in molecular cloning that allowed for high-throughput generation of complex targeting constructs (Angrand et al. 1999; Copeland et al. 2001; Lee et al. 2001; Valenzuela et al. 2003). Early proposals for a more systematic strategy advocated for a hybrid approach of chemical mutagenesis (e.g., ENU), gene trapping, and gene targeting for complete genome coverage and emphasized the need for adopting systematic phenotyping protocols, the development of databases to provide easy access to functional data, and animal repositories to ensure broad access to models and other key resources (Battey et al. 1999; Hrabe de Angelis et al. 2000; Nadeau et al. 2001; Nolan et al. 2000; Skarnes et al. 2004).

The Banbury meeting in 2003 further refined this concept to set forth a goal of creating a null, reporter allele for every gene as a foundational starting point for functional annotation (Austin et al. 2004). Despite deliberately flexible in the exact approach, the group adopted several key principles, including the use of a single inbred genetic background, and the open availability of cell, animal, and data resources to the scientific community. Generation of mice from these resources would follow at a pace that reflected both the community demand and capacity for centralized production, ultimately supporting individual investigator research programs. The concept aligned with European efforts, which emphasized the value of generating a conditional version of each allele (Auwerx et al. 2004). Together, these concepts were put into action as the NIH funded Knockout Mouse Project (KOMP), the European Conditional Mouse Mutagenesis Program (EUCOMM), and the Canadian-funded North American Conditional Mouse Mutagenesis Program (NorCOMM), which joined to form a singular effort as the International Knockout Mouse Consortium (IKMC) (Gondo 2008; International Mouse Knockout et al. 2007).

The bold IKMC mission of generating a null or conditional null allele for every gene in the mouse genome was complemented by the more modest goal of turning a small subset of these alleles into mouse strains for systematic characterization (Bradley et al. 2012; Skarnes et al. 2011). Further refinement of standard protocols for mouse phenotyping and the demonstration of the feasibility of large-scale mouse generation and phenotyping (de Angelis et al. 2015; White et al. 2013) underpin the current efforts of the International Mouse Phenotyping Consortium, which are now expanding on the initial vision first proposed at Banbury (Lloyd et al. 2020). The IMPC has generated over 5000 knockout lines from IKMC ES resources (Birling et al. 2021), and the advent of CRISPR/Cas9 technology has further augmented capacity for the generation of mutant mice. To date, the IMPC has generated knockout lines for 7590 genes (Data Release 14, May 7, 2021) and established a robust infrastructure sufficient for completing the draft functional annotation of a mammalian genome. This effort has had an enormous impact on our understanding mammalian biology and human disease (Brown et al. 2018; Meehan et al. 2017). The IMPC has continued to expand the catalog of mammalian essential genes, which are highly enriched in human disease (Cacheiro et al. 2020; Dickinson et al. 2016), while providing novel insights into developmental mechanisms through detailed embryonic phenotyping (Dickinson et al. 2016). The IMPC pipeline has revealed numerous novel gene associations with disease-relevant traits (Bowl et al. 2017; Rozman et al. 2018; Swan et al. 2020), and systematic phenotyping of both sexes reveals widespread sexual dimorphism (Karp et al. 2017).

What was once a long-term aspirational goal of the mouse genetics community is now an achievable milestone? This raises several interesting and important questions: How many more genes remain to be knocked out? How should these genes be prioritized for systematic mutagenesis? Are there other features in the genome (e.g., noncoding RNAs) that merit further consideration as mutagenesis targets? Here, we explore the collective catalog of mutant mouse null alleles generated by the IMPC and the broader scientific community to address these questions and chart a course towards finalizing a blueprint for understanding the activity of the protein-coding fraction of the mammalian genome.

## Results

### Current state of the mutant mouse null allele catalog to assess protein-coding gene function

**Fig. 2:**
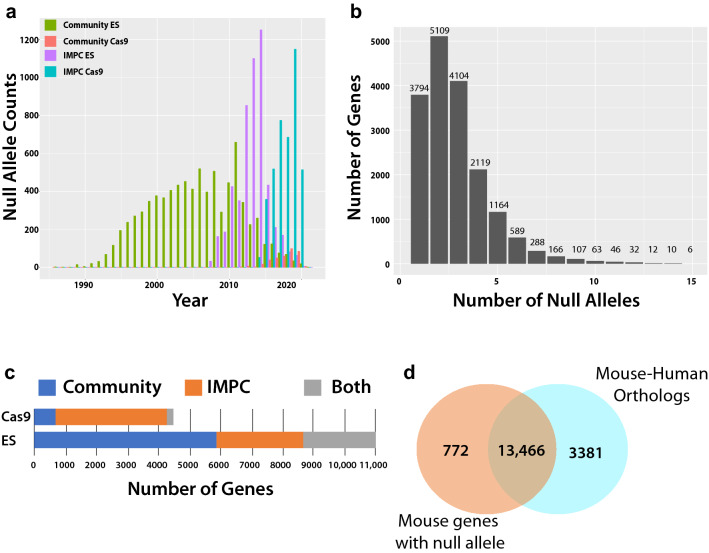
Summary of mouse null allele production and progress towards completing a resource encompassing mouse–human orthologs. **a** Overview of animal production using either ES-cell based technology or CRISPR/Cas9 within independent research groups representing the Community effort or as part of the International Mouse Phenotyping Consortium. Dates for unique alleles generated by the Community were determined using earliest citation for allele transmission and dates for IMPC alleles were based upon record of successful confirmation of germline line transmission (GLT). **b** Histogram for null allele counts per gene shows that most genes have 1–3 unique null alleles while another ~ 2000 have 5 or more null alleles. **c** Contributions of different technology employed by the Community and IMPC to generate the null allele. **d** Venn diagram comparing all mouse genes with a null allele to the total number of mouse genes with a high-confidence human ortholog

To focus on defining the community-wide effort to mutagenize the mouse genome, we include all null alleles, irrespective of their relationship to systematic production efforts or public availability. We define “IMPC” as all mouse model generation activities of the IKMC or IMPC, and “community” for all other alleles (individual investigator, non-IMPC programs, etc.). For curating null alleles, we used the MouseMine query tool to obtain annotation information detailing the target gene, allele symbol, methodology, and associated publications (Motenko et al. 2015). These queries identified 29,341 unique null alleles corresponding to 13,973 protein-coding genes with the majority of community-derived alleles generated using an ES-based resource and IMPC alleles a mixture of both ES and Cas9 derived. In addition to these targeted null mutations, we identified an additional 265 unique genes as having an annotated null allele resulting from random mutagenesis (spontaneous, ENU or gene trap). While our emphasis here is on the null resource, there are currently over 67,000 mutant mouse alleles documented by Mouse Genome Informatics (http://www.informatics.jax.org) that represent a diverse set of alleles ranging from hypomorphs to dominant negative, and humanized regions as well as others. This set of curated null alleles covers ~ 62% (14,238/23,000) of the protein-coding genes currently cataloged in the mouse genome. A detailed look at the time course for animal model generation highlights community production of null alleles rapidly accelerating in the mid-1990s as the technology spread and was implemented in core facilities to provide stable production of 400–500 new alleles published each year peaking in 2011 (Fig. 2a). About this time, IMPC production of null alleles ramped up significantly (Birling et al. 2021), which has been accompanied by a decline in community production of ESC-based alleles. This reduction may reflect the uptake of IKMC- and IMPC-generated resources by individual investigators, obviating the need to produce their own knockout. In 2015, the IMPC pivoted to the use of CRISPR/Cas9 to generate knockout alleles, which quickly replaced ESC-based animal production resulting in the establishment of ~ 4000 new lines within this 5-year window from 2015 to 2020 (Fig. 2a). Concurrently, there is a lower, but growing rate of community produced null alleles utilizing CRISPR/Cas9. Reflecting challenges in public availability of resources and parallel research aims, multiple null alleles are frequently generated for genes (Fig. 2b), including 150 genes with more than 10 null alleles. Most of community-generated null alleles are ESC based, while the vast majority of Cas9-generated null alleles reported in MGI were generated by the IMPC, due to rapid adoption of the technology as a core mutagenesis method to reduce cost and increase throughput (Fig. 2c).

Given the current efforts for complete functional annotation of the mammalian genome, with an overarching goal to understand human biology and disease, we examined how many mouse knockout alleles corresponded to protein-coding genes with high-confidence human orthologs defined by having multiple independent lines of supporting evidence from different resources (Munoz-Fuentes et al. 2018), and how many of these genes remain to be targeted. Of the 14,238 mouse genes that have a null allele, 94% (13,466) have a human ortholog while the remaining 772 lack a clearly identifiable ortholog or are mouse specific (Fig. 2d). Therefore, of the total 16,847 mouse genes with a high-confidence human ortholog, 79.9% have a reported null allele, leaving 3381 genes to complete the mutant mouse null resource.

**Fig. 3:**
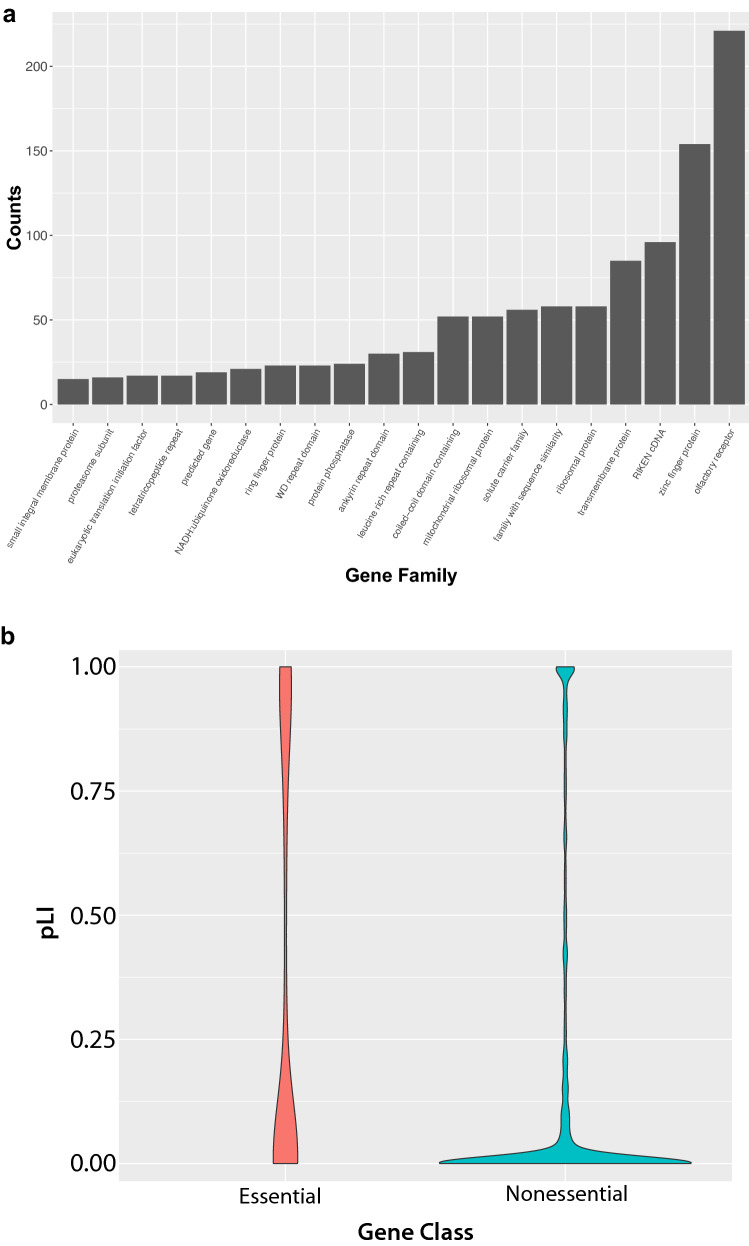
Analysis of gene family representation and constraint for the 3381 mouse–human orthologs that currently lack a null allele in mouse. **a** The set of remaining genes is specifically enriched for large gene families and the RIKEN cDNA collections. Olfactory receptors and zinc finger proteins are the most highly represented gene families. **b** Classification of the remaining genes using human orthologs to assess cell essentiality based upon CRISPR/Cas9 screens in cancer cell lines and functional constraint using probability of loss-of-intolerance (pLI) scores that range from 0 to 1 with values closer to 1 associated with higher level of constraint

To further develop a prioritization framework, we analyzed the remaining 3381 genes to determine if there were any gene families that were over-represented or if there was evidence of functional constraint on these genes lacking null alleles. Within this set of non-targeted genes, olfactory receptors were the most highly represented class of genes followed by zinc finger domain containing genes and RIKEN cDNA clones (Fig. 3a). This is consistent with large size of these gene families and highlights the barriers to research on genes for which there is little existing functional data (Stoeger et al. 2018). In addition, it is important to consider whether the information generated from knockout mice for a class of genes such as olfactory receptors is merited given the phenotyping tests included in the IMPC, and the expected impact of single gene mutation. Conversely, transmembrane proteins and solute carriers are more likely to be druggable and, thus, could be prioritized as gene families warranting completion. There are also many ribosome-related genes left to be characterized, and ribosomopathies are an emerging disease class that displays a wide range of phenotypes (Kampen et al. 2020). Additionally, genes essential for life are highly enriched for human disease genes (Bartha et al. 2018; Cacheiro et al. 2020; Dickinson et al. 2016; Georgi et al. 2013). Of the 3381 remaining genes, 15% (507/3381) are classified as cell essential based upon CRISPR/Cas9 screens in human cell lines (Cacheiro et al. 2020; Tsherniak et al. 2017). To determine if these genes were under constraint against mutation in the human population, we used the human orthologs to obtain the probability of loss-of-intolerance (pLI) score from the gnomAD database (Karczewski et al. 2020). pLI scores range from 0 to 1 with values closer to 1 indicative of significant constraint in the human genome (Lek et al. 2016). Of the cell essential genes, 144/507 have a human ortholog associated with a pLI score greater than 0.8 further supporting the idea that this set of cell essential genes is under functional constraint. In total, 11% (378/3381) of the genes without a null allele have a pLI score greater than 0.8 (Fig. 3b). Genes that are nonessential in cell lines but have high pLI scores have previously been shown to be enriched for critical developmental regulators that are also associated with human disease (Cacheiro et al. 2020).

**Fig. 4:**
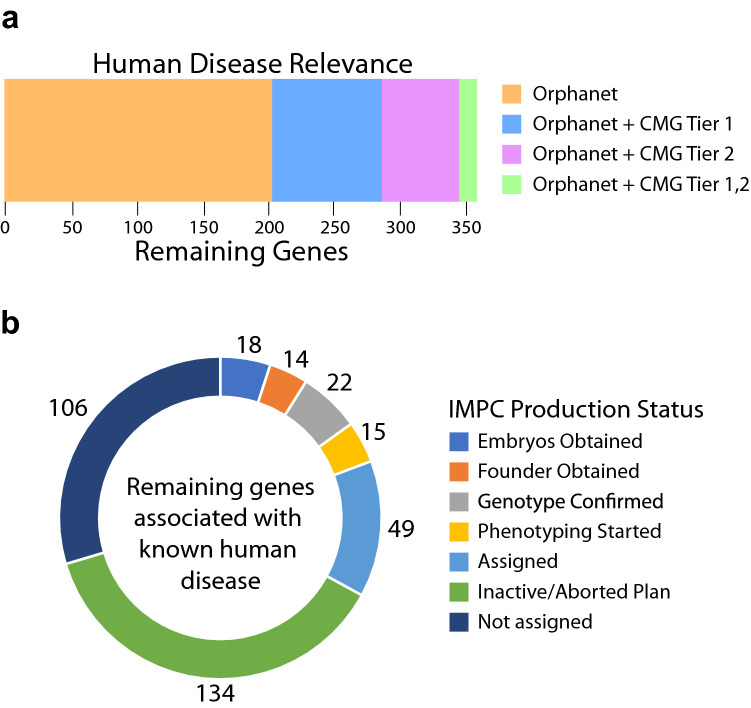
Potential human disease relevance for genes without a mouse null allele. **a** Human orthologs were used to query the Orphanet database (https://www.orpha.net/) and the Centers for Mendelian Genomics (CMG) gene lists (http://mendelian.org/phenotypes-genes). CMG genes are classified into Tier 1 and Tier 2 based upon the supporting level of evidence. Tier 1 genes have the highest-level of confidence with multiple levels of supporting evidence and Tier 2 genes are strong candidates but do not meet stringency criteria set for Tier 1. Currently, ~ 10% of the remaining mouse genes are related to human disease genes. **b** IMPC progress towards making knockouts for genes relevant to human disease. Of these 358 genes, 252 genes have either been previously attempted and failed (Inactive/aborted) or are currently assigned or in progress through the IMPC production and phenotyping pipeline

To determine the extent that genes without knockout alleles are related to human disease genes, we examined the overlap with ORPHANET (http://www.orpha.net) and Tier 1 (solved cases) and Tier 2 (unsolved cases) gene lists from the Centers for Mendelian Genomics (CMG; http://mendelian.org/phenotypes-genes) (Posey et al. 2019). This analysis highlighted 358 genes with support from ORPHANET-only or had additional evidence from the CMG (Fig. 4a). While these genes were identified as lacking a knockout mouse, the ongoing production efforts of the IMPC have made progress towards establishing knockouts for 54 genes and begun phenotyping on another 15 genes (Fig. 4b). Production attempts have failed for 134 genes with the remaining 106 yet to be targeted by the IMPC. It will be important to emphasize this set of human disease related genes for generation of mouse knockouts.

**Fig. 5:**
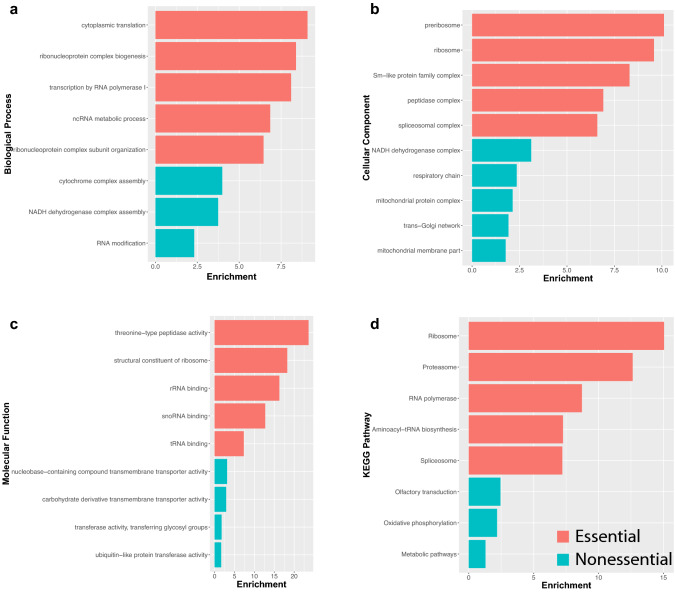
GO term enrichment and pathway analysis on the set of 3381 genes classified into cell essential and nonessential categories. GO terms for **a** Biological process, **b** Cellular component, and **c** Molecular function highlight a role for essential genes in core biological processes including transcription, splicing, and translation, and a role for nonessential genes in metabolic processes and mitochondrial function. **d** KEGG pathway analysis supports the distinction between cell essential and nonessential genes and associated biological function. Enrichment values were determined using WebGestalt with FDR < 0.5

Next, we performed Gene Ontology (GO) term enrichment (Fig. 5a–c) and KEGG pathway analysis (Fig. 5d) on the previously defined cell essential and nonessential gene sets using WebGestalt (Liao et al. 2019). Strikingly, these analyses revealed a stark distinction in the biological processes and pathways associated with these two groups of genes. Cell essential genes were significantly enriched (FDR < 0.05) for terms associated with pathways for mRNA splicing (spliceosome), translation (ribosome), and protein degradation (proteasome; Fig. 5a–d). In contrast, nonessential genes showed enrichment for olfactory transduction and metabolic pathways (Fig. 5d). These findings indicate a clear distinction between core biological functions required for cell survival versus potentially tissue-specific differences in energy requirements or activity of certain cell types. In support of these differences, we used the human orthologs for the remaining genes to identify potential human disease associations within OMIM and observed an enrichment for Mitochondrial Complex I Deficiency (*P*-value = 2.0673e−11), Alcohol Dependence (*P*-value = 6.5255e−4), and Mitochondrial Complex IV Deficiency (*P*-value = 4.4197e−5).

### Overview of noncoding transcripts annotated in the mouse genome

**Fig. 6:**
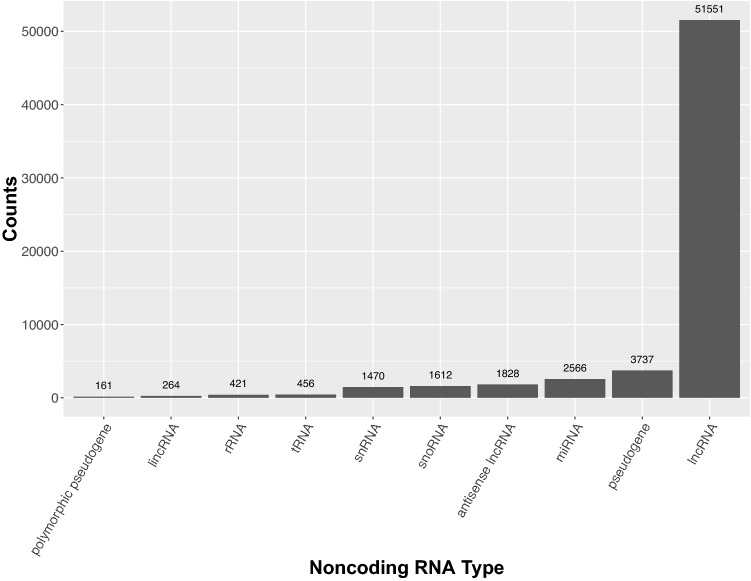
Top 10 noncoding RNA sequence features annotated in the mouse genome. The number of known long noncoding RNA’s (lncRNAs) currently exceeds the number of protein-coding genes and the vast majority remain to be studied in depth

Despite the intense focus on the protein-coding portion of the genome, there has been a long-standing appreciation for the role of noncoding transcripts for regulating diverse cellular events such as modulating chromatin structure (e.g., *Xist*), targeting genes to regulate expression (e.g., miRNA), and building the translational machinery (e.g., rRNA and tRNA). While these functions are known, the number of annotated noncoding transcripts continues to grow and now exceeds the number of protein-coding genes with the vast majority generically referred to as long noncoding RNA’s (lncRNAs) with unknown function (Fig. 6). To gain a greater understanding of how noncoding transcripts impact biology, we will need to invest and explore in alternative targeting strategies to determine if the noncoding transcript itself is functional or if it is merely the act of transcription that is required. Functional testing of the transcripts can be achieved using whole gene ablation strategies with CRISPR/Cas9; however, while large deletions (> 10 kb) are feasible with CRISPR/Cas9 these are often accompanied with other structural variants such as inversions and duplications which would require additional screening (Birling et al. 2017). Additionally, the removal of large genomic regions has the potential to impact the expression of neighboring genes by altering the *cis*-regulatory landscape. An alternative approach is to introduce a poly-adenylation sequence to terminate transcription (Engreitz et al. 2016) or knockdown the target the transcript by introducing a degradation sequence or using Cas13a (Gao et al. 2020). These approaches would keep the locus intact but prevent the transcript from accumulating; and thereby, determine if the transcript itself is functional or is it merely the engagement of RNA polymerase II that is required. There is also a pre-existing set of ESC-based resources available for studying noncoding RNA function (Hansen et al. 2021). Our current animal production methodologies are well suited to be scaled to address these questions and can readily implement all of these approaches.

## Conclusions

In summary, extraordinary progress has been made towards generating the first complete functional annotation of the protein-coding fraction of the mammalian genome. This celebratory milestone is rapidly approaching thanks to the combined efforts of the research community and the ongoing IMPC initiative. For the remaining genes, strategic implementation of different prioritization strategies will allow us to focus on sets of genes that have the potential to inform on human disease, increase our understanding of the broad biological function of large gene families, and shed light on the darkest parts of the genome. Thus far, in IMPC-generated strains, the rate of gene essentiality has remained constant at ~ 35% where 25% of lines are classified as lethal and an additional 10% are classified as subviable (Cacheiro et al. 2020; Dickinson et al. 2016). It will be informative to determine whether these rates will remain stable for this gene set given the relative dearth of functional information available for these genes. Elucidating the full spectrum of gene essentiality within mammals has the potential to further support ongoing human disease gene discovery efforts and to provide new insights into the underlying mechanisms of congenital birth defects and later onset diseases that have a developmental etiology. Our initial characterization of the production attempts for these remaining genes suggests that some may be refractory to our current methodologies possibly due to gene essentiality or haploinsufficiency and, thus, may require a more nuanced approach. Conditional alleles could provide a means to circumvent production challenges and would provide useful tools for further mechanistic investigation of essential gene function. Alternatively, novel approaches such as the implementation of auxin-inducible protein degradation may help to overcome null allele production efforts (Yesbolatova et al. 2020). Further, the classification of a null allele is based upon the assumption that frameshift mutations will result in mRNA degradation via the nonsense-mediated decay (NMD) pathway. While the IMPC design principles adhere to well-established rules of NMD, recent work has shown that there are a number of exceptions to the canonical rules, and that roughly 25% of the variance from expected NMD remains unexplained (Lindeboom et al. 2016). Moreover, there are reported cases of exon skipping as well as the use of alternate downstream translation start sites that allow for some level of activity (Makino et al. 2016). The extent to which these apply to the current catalog will require further bioinformatic and experimental determination. As mentioned above, the rate of gene essentiality has remained constant for IMPC-generated lines regardless of the technology used to generate the allele. Thus, as the foundation for a phenotype-driven screen, the mutant alleles characterized to date significantly disrupt gene function.

In addition, the generation of alleles for precision medicine corresponding to patient-specific mutations will undoubtedly be a critical next step to further our understanding of the molecular basis of human disease. Beyond the coding genome, the vast majority of noncoding RNAs have yet to be characterized in mutant mice. The collective effort to generate a complete null resource will provide a strong foundation to support these future initiatives, expanding our understanding of genome function, which holds great promise for improving human health and personalized medicine.
